# A Case of Oral-Buccal-Lingual Dyskinesia and Neuropsychiatric Symptoms After Prolonged Levetiracetam Exposure

**DOI:** 10.7759/cureus.62692

**Published:** 2024-06-19

**Authors:** Kamalakar Surineni, Vy Le, Danielle Jones

**Affiliations:** 1 Psychiatry and Behavioral Sciences, University of Kansas Medical Center, Wichita, USA; 2 Psychiatry and Behavioral Sciences, University of Kansas School of Medicine, Wichita, Wichita, USA

**Keywords:** td, levetiracetam, tardive dyskinesia, dyskinesia, oral-buccal-lingual dyskinesia

## Abstract

Tardive dyskinesia (TD) is a serious and often permanent complication usually seen after the long-term use of antipsychotic medications, and multiple other classes of medications have been reported to cause TD or TD-like syndromes. TD can affect any part of the body, but it most commonly affects the mouth, lips, and tongue. We present a case of oral-buccal-lingual dyskinesia in an 86-year-old female from the long-term use of levetiracetam for a seizure disorder. The patient was started on levetiracetam four years before admission and was noted to have an acute onset of oral-buccal-lingual dyskinesia that was so severe it interrupted the patient’s speech and feeding. The patient’s dyskinesias are completely resolved after cross-tapering levetiracetam 500 mg twice a day with valproic acid 750 mg daily. Additionally, there was a global recovery of the patient’s mood and psychosis after the cross-taper. Our case highlights the potential implications of levetiracetam in dyskinetic movements and neuropsychiatric symptoms, and it warrants close monitoring of patients taking this medication especially elderly with multiple comorbidities and compromised renal function. Moreover, the case suggests the reversible nature of both neuropsychiatric symptoms and dyskinesias.

## Introduction

Tardive dyskinesia (TD) are abnormal, involuntary movements of the tongue, jaw, trunk, or extremities associated with the chronic use of dopamine receptor-blocking drugs (DRBDs). Per the Diagnostic and Statistical Manual of Mental Disorders-V-Text Revision (DSM-V-TR) published in 2022, there must be a history of the use of the offending agent for at least three months (or one month in individuals of 60 years or older) and the movements should be present over at least four weeks [1]. TD can affect any part of the body, but it most commonly affects the mouth, lips, and tongue.

Antipsychotic drugs are most associated with TD, with an estimated prevalence of 20-30% among patients taking these medications [2]. Other drug classes, including those with no dopaminergic activity, have also been linked to TD, although mainly in case reports or series [3]. These include antidepressants [4], antiepileptics [5], anticholinergics [6], and calcium channel blockers [7].

Although generally well-tolerated, antiepileptic drugs such as levetiracetam have rarely been associated with various movement disorders such as tremors, ataxia, and dyskinesias [8,9]. We present a case of oral-buccal-lingual dyskinesia and neuropsychiatric symptoms in an elderly female with a long history of levetiracetam-treated seizure disorder that resolves after the discontinuation of levetiracetam.

This case report highlights the potential link between the long-term use of antiepileptic drugs, particularly levetiracetam, and the development of TD. It underscores the importance of recognizing and managing the side effects of these medications promptly.

## Case presentation

An 86-year-old African American female with a complex medical history was admitted to the emergency department with chest pain, shortness of breath, fatigue, and dark stool. Her past medical history included refractory hypertension, chronic anemia, chronic kidney disease, coronary artery disease, heart failure with preserved ejection fraction, chronic myeloid leukemia in remission, and seizure disorder. She was taking multiple medications for these conditions; please refer to Table 1 for the list of the patient's medications at the time of admission. The patient used to be on phenytoin but was switched to levetiracetam around four years ago after she was incidentally found to have high phenytoin levels.

**Table 1:** List of medications the patient was taking at the time of admission.

Medications	Dosage
Allopurinol	300 mg daily
Calcitriol	0.5 mcg daily
Carvedilol	25 mg twice a day
Clonidine	0.2 mg three times a day
Clopidogrel	75 mg daily
Escitalopram	5 mg daily
Furosemide	40 mg daily
Gabapentin	300 mg twice a day
Iron	325 mg twice a day
Levetiracetam	500 mg twice a day
Levothyroxine	100 mcg daily
Nifedipine	90 mg daily
Pantoprazole	40 mg daily

Upon admission, she was found to have an acute-on-chronic kidney injury, bleeding duodenal arteriovenous malformations, and community-acquired pneumonia. She received treatment including antibiotics and a blood transfusion. However, she developed acute agitation and altered mental status, leading to a consultation with psychiatry.

**Figure 1:**
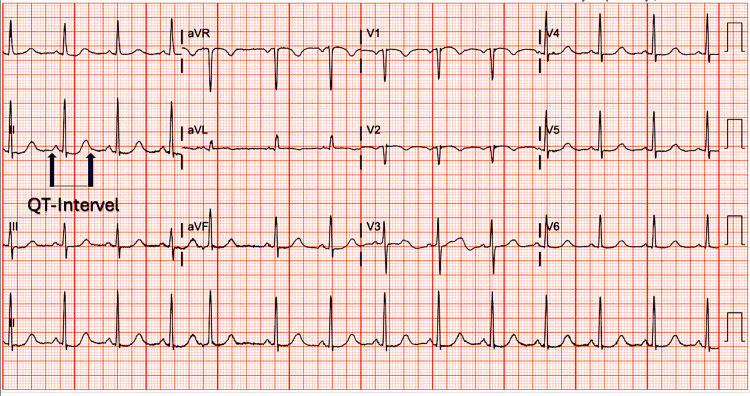
Patient's ECG showing QTc prolongation of 493 (reference range for females is 360-460). ECG: Electrocardiogram; QTc: Corrected QT Interval

The patient received haloperidol 0.5 mg two doses and one dose of quetiapine 25 mg at bedtime in the first three days of admission for agitation, but both were later discontinued because of corrected QT (QTc) prolongation as noted in the ECG (Figure 1).

**Figure 2:**
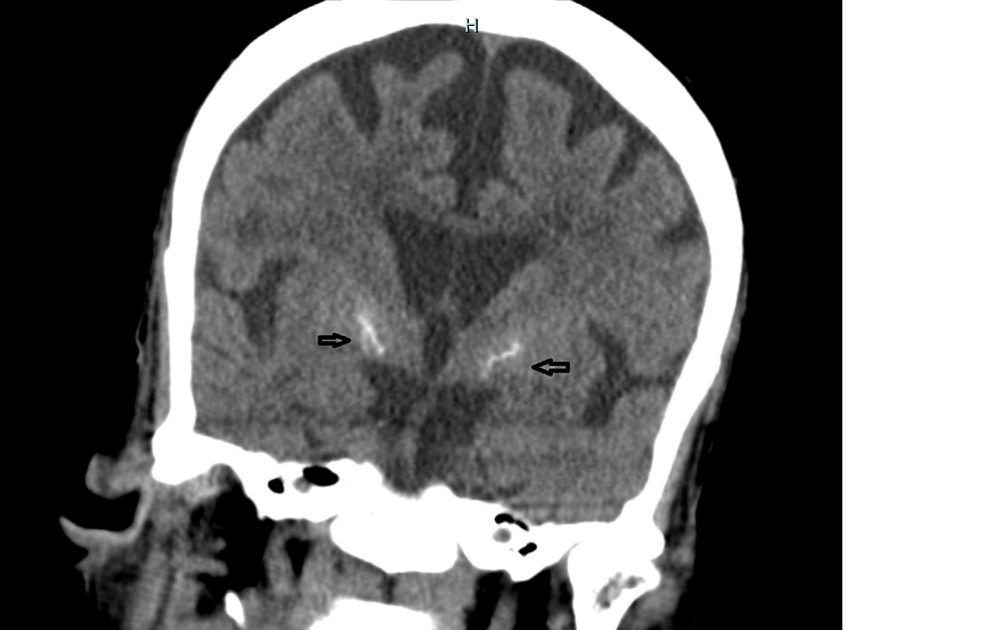
CT scan of the head without contrast showing calcifications within her basal ganglia and generalized atrophy with involutional changes.

Diagnostic workup, including vitamin B12 and folate levels, thyroid-stimulating hormone, HIV, and rapid plasma regain (RPR), was unremarkable. Brain CT showed no acute findings but revealed calcifications within her basal ganglia, microvascular ischemic changes, and generalized atrophy with involutional changes (Figure 2).

She was medically stabilized before being transferred to the senior behavioral health unit (SBHU) on hospital day 13.

On evaluation at the SBHU, she exhibited choreoathetosis of the tongue, described as repetitive, irregular, nonrhythmic tongue protrusion, and lip-smacking, which was a new development. The dyskinesias are severe enough to interfere with speech and eating. Along with dyskinesia, she has psychomotor agitation, combativeness, active hallucinations, and delusional thinking. Given the potential implications of levetiracetam in her symptoms, the medication was cross-titrated to divalproex sodium 750 mg (serum trough level: 77 mcg/mL). A steady reduction and complete disappearance of abnormal involuntary movements and agitation were noted after completing the cross-titration.

## Discussion

Levetiracetam is commonly prescribed because of its broad spectrum efficacy for various types of seizures and relative tolerability. However, it is associated with well-documented neuropsychiatric side effects. The highest rates of psychiatric and behavioral side effects for levetiracetam were reported in a review of 4,085 patients, with a rate of 22.1% compared to other antiepileptic drugs [10]. Despite being outlined in the medication’s package insert related to post-marketing experience [11], there is limited literature on the dyskinetic effects of levetiracetam. One prior case report described levetiracetam-induced chorea in a 28-year-old woman with seizures from brain metastasis attributed to spinal cord glioblastoma, which resolved upon switching from levetiracetam to a combination of lorazepam and phenytoin [8], and there is also contrary evidence where levetiracetam was effective in reducing TD that was developed from chronic neuroleptic use [9]. Elderly or people with preexisting mental illnesses may be more prone to neuropsychiatric side effects including dyskinesia from levetiracetam, but there is no evidence necessitating further research.

Various theories exist about the pathogenesis of TD, including chronic exposure to drugs causing upregulation of dopamine receptors and dysfunctional gamma-aminobutyric acid (GABA) neurons leading to an imbalance in basal ganglia pathways [12,13]. Neurodegenerative changes to the basal ganglia may increase susceptibility [14] to TD, as demonstrated in our patient with basal ganglia calcifications noted on CT (Figure 1). Levetiracetam's inhibition of synaptic vesicle glycoprotein 2A and alteration of GABA metabolism and turnover in the striatum may contribute to dyskinesia by influencing striatal dopamine release [15].

Our patient had a previous episode of involuntary movement when gabapentin was missed, suggesting a potential link between GABAergic agents and dyskinesia. Despite a recent meta-analysis negating a relationship between levetiracetam dose and adverse effects [16], there are independent case reports highlighting dose-dependent neuropsychiatric effects [17] including worsening depression and suicidal behaviors [18], and aggression [19]. Genetic variants, gender, underlying cognitive impairment, hypertension, and microvascular ischemic changes (Figure 1) are known risk factors for TD [3].

It is important to consider alternative explanations for tongue dyskinesia, such as spontaneous dyskinesia, movement disorders secondary to renal failure, and subclinical seizures, which may have been undetected without EEG monitoring. Although the dose and frequency of haloperidol or quetiapine are insufficient to cause TD, it may still need to be considered as a risk factor.

## Conclusions

The patient in this case showed a significant improvement in dyskinetic movements and psychiatric symptoms after switching from levetiracetam to valproic acid. Patients may have developed these adverse effects from the chronic use of levetiracetam or precipitated by acute-on-chronic renal failure, and the elderly or people with preexisting mental illnesses may be more prone to neuropsychiatric side effects including dyskinesia, but more research is needed to prove this association. This case highlights the potential implications of levetiracetam in neuropsychiatric symptoms and dyskinetic movements and warrants close monitoring of patients taking this medication, especially the elderly with multiple comorbidities and compromised renal function. Our case also suggests the reversible nature of dyskinetic movements and neuropsychiatric symptoms.
